# Effects of the COVID-19 Pandemic on Anti-vascular Endothelial Growth Factor Treatment in China

**DOI:** 10.3389/fmed.2020.576275

**Published:** 2020-12-14

**Authors:** Kai-Bo Yang, Hao Feng, Han Zhang

**Affiliations:** Department of Ophthalmology, The First Hospital of China Medical University, Shenyang, China

**Keywords:** COVID-19, anti-VEGF (vascular endothelial growth factor), age-related macula degeneration (AMD), retinal vein occlusion (RVO), diabetic macular edema (DME)

## Abstract

We evaluated the impact of the COVID-19 pandemic on anti-VEGF treatment in ophthalmology patients in a single hospital in northern China. A total of 93 anti-VEGF injections were administered to 85 eyes of 72 patients at The China Medical University First Hospital Department of Ophthalmology during the COVID-19 pandemic. Compared to the same period in 2019, the number of injections decreased by 70%. Fifty-nine eyes of 46 patients were receiving 3+PRN anti-VEGF treatment prior to the outbreak of the COVID-19 pandemic; all of these patients experienced treatment interruptions due to COVID-19-associated reasons. Anatomic and functional outcomes suggest that patients with anti-VEGF treatment interruptions are at risk for severe adverse visual sequelae. Moreover, deferred anti-VEGF treatment due to patient-related or department-related reasons during the COVID-19 pandemic may result in poor visual outcomes for new patients. Our results suggest that COVID-19 has had a significant negative effect on anti-VEGF treatment in ophthalmology patients. Detailed guidance from global experts in ophthalmology is highly sought after in these challenging circumstances.

## Introduction

The outbreak of the coronavirus disease 2019 (COVID-19) pandemic was sudden and tragic. It has also been massively disruptive to the practice of ophthalmology. The impact has affected several areas, including the shortage of personal protective equipment required by clinics to prevent possible infection of patients and staff. Outpatient and surgical volumes have decreased (in some instances by >75%) during the pandemic, and treatment has been restricted to urgent or emergency conditions (1). A serious consequence of the decreased volumes is that many patients are experiencing irreversible loss of sight. Intravitreal injection of anti-vascular endothelial growth factor (VEGF) agents is widely regarded as the standard of care for patients with retinal disease, including neovascular age-related macular degeneration (nAMD), diabetic macular edema (DME), and macular edema (ME) due to retinal vein occlusion (RVO), and has become the most commonly performed ophthalmic procedure. This study aimed to evaluate the impact of the COVID-19 pandemic on anti-VEGF treatment in ophthalmology patients in a single hospital in northern China.

## Methods and Results

**Figure 1:**
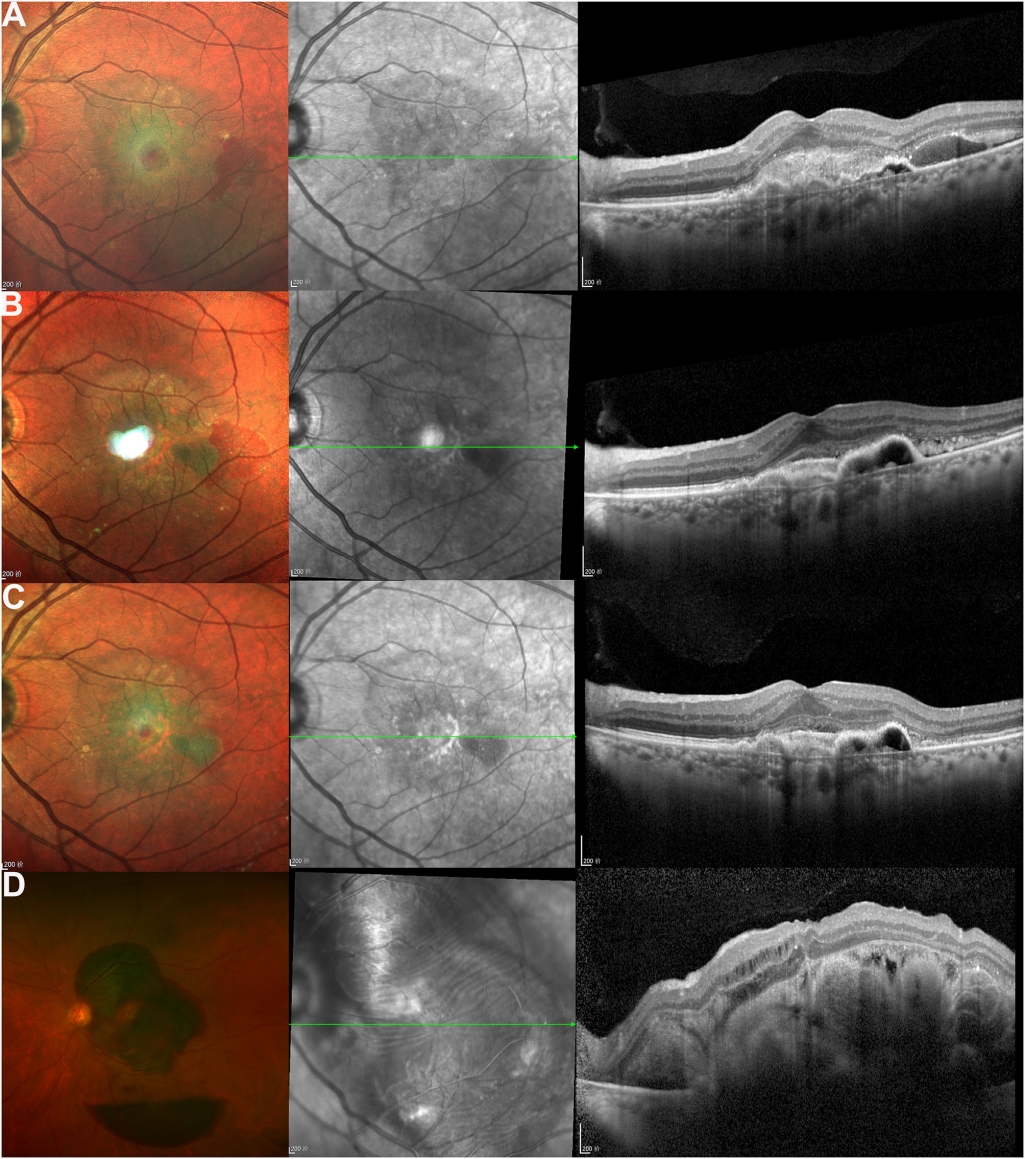
The images were obtained from a 67-year-old male patient with neovascular age-related macular degeneration (nAMD) in the left eye. The patient's best-corrected visual acuity (BCVA) was 20/63 at baseline. The scanning laser ophthalmoscopy (SLO) image at baseline **(A)** shows a subfoveal lesion and subretinal hemorrhage. The optical coherence tomography (OCT) image shows type I neovascularization (NV), as well as subretinal hyperreflective material (SHRM) and subretinal accumulation of fluid. The patient received Ranibizumab injections with a 3+PRN treatment regimen prior to the outbreak of the COVID-19 pandemic. At the 1- and 2-month follow-ups, the patient's BCVA improved to 20/40 and 20/32, respectively. The follow-up SLO and OCT images after 1 month **(B)** and 2 months **(C)** show the resolution of subretinal hemorrhage and SHRM. The patient then experienced a 4.5-month interruption in their treatment due to a COVID-19-associated reason (patient concerns). When the patient returned for the 6.5-month follow-up, their BCVA decreased to counting fingers. The ultra-widefield retinal image **(D)** shows extensive subretinal hemorrhage and serous-hemorrhagic pigment epithelial detachment (PED) near the inferotemporal vascular arcade. The OCT image shows massive SHFM and intraretinal edema.

We retrospectively reviewed the charts of all patients who received anti-VEGF treatment in The China Medical University First Hospital Department of Ophthalmology from January 21, 2020 (the day on which the outpatient services and operations were restricted because of the pandemic), to June 1, 2020. A total of 85 eyes of 72 patients received 93 anti-VEGF injections during the study period, including 35 eyes (29 patients) with nAMD, 17 eyes (10 patients) with DME, 15 eyes (15 patients) with central RVO (CRVO)-ME, five eyes (five patients) with branch RVO (BRVO)-ME, and 13 eyes (13 patients) with proliferative diabetic retinopathy (PDR, anti-VEGF injection as an adjuvant treatment before vitrectomy). Eight eyes (six patients) with nAMD received injections on two occasions. Compared to the same period in 2019 (307 anti-VEGF injections in 199 eyes of 185 patients), the number of injections decreased by 70% during the COVID-19 pandemic. After excluding eyes with PDR that were receiving anti-VEGF injection as an adjuvant treatment before vitrectomy, the mean ± standard deviation (SD) logarithm of minimal angle of resolution (logMAR) best-corrected visual acuity (BCVA) at the last follow-up before the COVID-19 pandemic was 0.59 ± 0.22 (20/78 in Snellen equivalent). At the last follow-up during the COVID-19 pandemic, mean BCVA decreased significantly to 0.86 ± 0.41 (20/145 in Snellen equivalent) (*P* < 0.001, Wilcoxon signed-rank tests), which compared poorly with that during the same period in 2019 (Table 1). In 2019, the mean BCVA at the equivalent time of the last follow-up was 0.66 ± 0.39 (20/91 in Snellen equivalent) and during the same period as the pandemic, it was 0.53 ± 0.36 (20/68 in Snellen equivalent). Fifty-nine eyes (81.9%) (46 patients) were already undergoing a 3+PRN anti-VEGF treatment regimen prior to the outbreak of the COVID-19 pandemic, and all of these patients experienced an interruption in their treatment due to COVID-19-associated reasons, such as travel restrictions, patient concerns, and department-related reasons (i.e., appointments for further follow-ups or injections could not be adequately scheduled). During the same period in 2019, only 32 eyes (21.5%) experienced treatment interruption duration of >4.5 months. The clinical characteristics of patients with treatment interruption are presented in Table 2. Before treatment interruption, these eyes had been treated for an average of 7.4 ± 7.8 months (range, 1–32 months), with a mean of 4.3 ± 3.1 injections (range, 1–15 injections). Snellen best-corrected visual acuity (BCVA) before treatment interruption ranged from 20/400 to 20/32, with a median of 20/63. The mean ± SD logMAR BCVA was 0.57 ± 0.23 (20/74 in Snellen equivalent). The mean ± SD central retinal thickness (CRT) was 358.7 ± 164.3 μm before treatment interruption. The mean length of treatment interruption was 5.3 ± 0.8 months (range, 4.5–7 months). On the return visit after treatment interruption, the mean ± SD logMAR BCVA had worsened significantly to 0.98 ± 0.41 (Snellen equivalent of 20/191) compared with the baseline value (*P* < 0.001, Wilcoxon signed-rank tests). Thirty-nine eyes (66.1%) lost ≥3 BCVA lines, with two eyes having a final BCVA of Hand Motion (HM) or worse, including 20 eyes (70%) with nAMD, 10 eyes (66.7%) with DME, seven eyes (70%) with CRVO-ME, and two eyes (50%) with BRVO-ME. There was a statistically significant correlation between decreases in the mean logMAR BCVA and increases in the length of the treatment interruption (Pearson's correlation analysis; *r* = 0.386, *P* = 0.003). In the multivariate analysis (stepwise linear regression analysis), longer treatment interruption was associated with worsened visual acuity (*P* = 0.003) (Table 3). On the return visit after treatment interruptions, all eyes exhibited more pronounced ME compared with their “before treatment interruption” evaluation. The mean ± SD CRT significantly increased to 608.1 ± 239.3 μm compared with that before treatment interruption (*P* < 0.001, Wilcoxon signed-rank tests). Seven eyes developed neovascular complications, including five eyes with DME and one eye with CRVO-ME developed neovascularization on the disc or neovascularization elsewhere, and one eye with CRVO-ME developed neovascular glaucoma. An example of the fundus and OCT images in a nAMD patient who experienced treatment interruption is shown in Figure 1.

**Table 1:** Clinical characteristics and visual acuity outcomes of patients receiving anti-VEGF treatment during the COVID-19 pandemic and the same period in 2019.

**Feature**	**COVID-19 pandemic**	**Same period in 2019**	***P*-value**
Age (yrs), (range)	62.4 ± 12.0 (29–89)	64.7 ± 13.2 (33–83)	0.426
Sex, *n* (%)			0.756
Male	32 (54.2)	71 (51.8)	
Female	27 (45.8)	66 (48.2)	
Eye conditions, *n* (%)			0.043
nAMD	35 (48.7)	54 (36.2)	
DME	17 (23.6)	39 (26.2)	
CRVO	15 (20.8)	25 (16.8)	
BRVO	5 (6.9)	31 (20.8)	
Eyes with treatment interruption duration ≥4.5 m, *n* (%)	59 (81.9)	32 (21.5)	< 0.001
BCVA at last follow-up before the study period (logMAR)	0.59 ± 0.22	0.66 ± 0.39	0.349
BCVA at last follow-up during the study period (logMAR)	0.86 ± 0.41	0.53 ± 0.36	< 0.001

*The eyes with proliferative diabetic retinopathy receiving anti-VEGF injection as an adjuvant treatment before vitrectomy were excluded*.

*The data are presented as the mean ± SD where applicable*.

*BCVA, best-corrected visual acuity; BRVO, branch retinal vein occlusion; CRT, central retinal thickness; CRVO, central retinal vein occlusion; DME, diabetic macular edema; LogMAR, logarithm of minimal angle of resolution; nAMD, neovascular age-related macular degeneration; SD, standard deviation*.

**Table 2:** Clinical characteristics of patients with treatment interruption.

**Feature**	**Number**
Age (yrs), mean ± SD (range)	63.4 ± 11.4 (29–76)
**Sex**, ***n*** **(%)**	
Male	26 (56.5)
Female	20 (43.5)
**Eye conditions**, ***n*** **(%)**	
nAMD	30 (50.8)
DME	15 (25.4)
CRVO	10 (16.9)
BRVO	4 (6.8)
Injections before treatment interruption, mean ± SD (range)	4.3 ± 3.1 (1–15)
Treatment length before treatment interruption (months), mean ± SD (range)	7.4 ± 7.8 (1–32)
BCVA before treatment interruption (logMAR), mean ± SD	0.57 ± 0.23
CRT before treatment interruption (μm), mean ± SD	358.7 ± 164.3
Treatment interruption length (months), mean ± SD (median)	5.3 ± 0.8 (5)
BCVA on return visit (logMAR), mean ± SD	0.98 ± 0.41
CRT on return visit(μm), mean ± SD	608.1 ± 239.3
**Complication on return visit**, ***n***	
NVD or NVE	6
Neovascular glaucoma	1

*BCVA, best-corrected visual acuity; BRVO, branch retinal vein occlusion; DME, diabetic macular edema; CRT, central retinal thickness; CRVO, central retinal vein occlusion; LogMAR, logarithm of minimal angle of resolution; nAMD, neovascular age-related macular degeneration; SD, standard deviation; NVD, neovascularization of the disc; NVE, neovascularization elsewhere*.

**Table 3:** Association between baseline characteristics and best-corrected visual acuity at final visit after treatment interruption.

**Baseline characteristics**	***P*-value**	**β**	**95% confidence intervals**
Age (yrs)	0.927		
Sex, male:female	0.581		
Eye conditions	0.149		
Number of injections before treatment interruption	0.576		
Treatment length before treatment interruption (months)	0.205		
BCVA before treatment interruption (logMAR)	0.795		
CRT before treatment interruption (μm)	0.430		
treatment interruption length (months)	0.003	0.386	0.060–0.266

*BCVA, best-corrected visual acuity; CRT, central retinal thickness; LogMAR, logarithm of minimal angle of resolution*.

## Discussion

Our results suggest that COVID-19 has had a significant and negative effect on anti-VEGF treatment of ophthalmology patients in a single hospital in northern China. The maintenance of a normative, adequate treatment course is very important for anti-VEGF therapy, especially in patients with nAMD (2). Anti-VEGF therapy for patients with CRVO-ME also requires ongoing, perpetual treatment in the majority of eyes (3). Patients with long-term treatment interruption are at risk for severe adverse visual sequelae (4, 5). Moreover, deferred anti-VEGF treatment due to patient-related or department-related reasons during the COVID-19 pandemic will also lead to poor visual outcomes in new patients. The Royal College of Ophthalmologists has developed guidelines for patients receiving anti-VEGF treatment during the COVID-19 pandemic (6). However, these guidelines are specifically relevant to the UK healthcare system, and their application outside of the UK is confounded by local regulations, practice capacities, and other country-specific factors. Management of patients receiving anti-VEGF injections during the COVID-19 pandemic will require changes to regular clinical practice to minimize the risk of exposure for patients and healthcare staff and to prioritize those patients with the greatest medical need.

Our study has several limitations. Given the retrospective nature, selection bias is anticipated. Moreover, our small sample size also limits the power of the analysis. Many patients lost to follow-up never returned, which is problematic because we are unable to determine the treatment outcomes for these patients. To determine the true sequelae in patients who do not follow up with anti-VEGF therapy, future studies that reach out to patients who do not return are needed. However, the strength of our study is that we could demonstrate the real-world impact of the COVID-19 pandemic on intravitreal injection practices and the effect on visual acuity in patients receiving anti-VEGF treatment. In line with previous studies (4, 7, 8), our results showed that unintentional treatment interruptions can result in remarkable deterioration of visual acuity. As visual acuity outcomes may be dependent on the length of treatment interruption (8), a long-term study may provide more information. Our results suggest that more detailed guidance from global medical retina experts is highly sought after during the challenging circumstances of the COVID-19 pandemic.

## Data Availability Statement

The original contributions presented in the study are included in the article/Supplementary Materials, further inquiries can be directed to the corresponding author/s.

## Ethics Statement

The studies involving human participants were reviewed and approved by Ethical Committee and the Institutional Review Board of China Medical University. The patients/participants provided their written informed consent to participate in this study.

## Author Contributions

HZ: conceptualization, writing—original draft preparation, and funding acquisition. K-BY and HZ: formal analysis. K-BY, HF, and HZ: investigation and data curation. All authors contributed to the article and approved the submitted version.

## Conflict of Interest

The authors declare that the research was conducted in the absence of any commercial or financial relationships that could be construed as a potential conflict of interest.
