# Supplementary material for: Effects of the COVID-19 Pandemic on Anti-vascular Endothelial Growth Factor Treatment in China
Source: Front Med (Lausanne). 2020 Dec 14;7:576275. doi: 10.3389/fmed.2020.576275 (PMC7768079; doi:10.3389/fmed.2020.576275)
Supplement: Supplementary file 1 [file Table_1.DOCX]

**Clinical Characteristics of Patients Maintained their Visual Acuity after Treatment Interruption**

| Feature | P 1 | P 2 | P 3 | P 4 |
| --- | --- | --- | --- | --- |
| Age (yrs) | 71 | 75 | 67 | 72 |
| Sex | M | F | M | M |
| Eye condition | nAMD | nAMD | nAMD | BRVO |
| Injections before treatment interruption | 11 | 5 | 7 | 4 |
| Treatment length before treatment interruption (months) | 15 | 8 | 11 | 5 |
| BCVA before treatment interruption (logMAR) | 1.0 | 1.0 | 0.8 | 0.5 |
| CRT before treatment interruption (μm) | 321.5 | 399.7 | 360.4 | 240.6 |
| Treatment interruption length (months) | 7 | 5 | 5.5 | 5 |
| BCVA on return visit (logMAR) | 1.0 | 1.0 | 0.8 | 0.6 |
| CRT on return visit(μm) | 398.2 | 421.9 | 389.3 | 299.5 |
| Visual acuity maintained was defined as visual acuity decrease < 2 lines.  BCVA= best-corrected visual acuity; BRVO= branch retinal vein occlusion; CRT= central retinal thickness; LogMAR= logarithm of minimal angle of resolution; nAMD= neovascular age-related macular degeneration; P= patient. | | | | |
